# Hexasodium Fytate (SNF472 or CSL525) Inhibits Ectopic Calcification in Various Pseudoxanthoma Elasticum and Calcinosis Cutis Animal Models

**DOI:** 10.3390/ph18040567

**Published:** 2025-04-14

**Authors:** Miguel D. Ferrer, Maria del Mar Pérez-Ferrer, Marc Blasco, Ida Joely Jacobs, Qiaoli Li, Olivier M. Vanakker, Lisa Dangreau, Andrea López, Gianluca Malagraba, Firas Bassissi, Joan Perelló, Carolina Salcedo

**Affiliations:** 1 Renal Lithiasis and Pathological Calcification Group (LiRCaP), Research Institute of Health Sciences (IUNICS), University of the Balearic Islands, 07122 Palma, Spain; miguel-david.ferrer@uib.es (M.D.F.); mmar.perez@uib.es (M.d.M.P.-F.); marc.blasco@uib.es (M.B.); gianluca.malagraba@uib.es (G.M.); 2 Health Research Institute of the Balearic Islands (IdISBa), 07010 Palma, Spain; 3 Sanifit Therapeutics S.A., a company of CSL Vifor, 07121 Palma, Spain; 4 Molecular Biology, Health Geography and One Health (MolONE), University of the Balearic Islands, 07122 Palma, Spain; 5 Department of Biochemistry and Molecular Biology, PXE International Center of Excellence in Research and Clinical Care, Thomas Jefferson University, Philadelphia, PA 19107, USA; ida.joely.jacobs@jefferson.edu (I.J.J.); qiaoli.li@jefferson.edu (Q.L.); 6 Center for Medical Genetics, Ghent University Hospital, 9000 Ghent, Belgium; olivier.vanakker@ugent.be (O.M.V.); lisa.dangreau@ugent.be (L.D.); 7 International Network on Ectopic Calcification (INTEC), 9000 Ghent, Belgium

**Keywords:** calcification inhibitors, calciphylaxis, ectopic calcification, inositol hexaphosphate, myo-inositol hexakisphosphate, phytate, vascular calcification

## Abstract

**Background/Objectives:** Ectopic calcification is a pathological condition characterized by the mineralization of soft tissues due to the deposition of calcium phosphate crystals. Hexasodium fytate (CSL525, previously known as SNF472) is a crystallization inhibitor being developed for the treatment of ectopic calcification-related disorders. Our aim was to investigate CSL525 for the treatment of soft-tissue calcification disorders in animal models of pseudoxanthoma elasticum and calcinosis cutis. **Methods:** In a first study, *abcc6^-/-^* zebrafish larvae were exposed to 1 mM CSL525 for 7 days or kept under the same conditions without CSL525, and spinal mineralization was quantified. In a second study, *abcc6^-/-^* mice were administered subcutaneously with CSL525 at 15 mg/kg thrice weekly for eight weeks. Vehicle-treated WT (C57BL/6J) and *abcc6^-/-^* mice served as controls, and muzzle skin calcification was quantified. In a third study, calcinosis cutis was induced in rats through subcutaneous administration of 0.15 mg FeCl_3_ at two sites in the thorax. Rats were administered either subcutaneous CSL525 (60 mg/kg) or vehicle (0.9% NaCl), and calcium content was measured in the skin. **Results:** CSL525 significantly reduced the calcified area (~40%) in *abcc6a^-/-^* zebrafish larvae. The *abcc6^-/-^* mice receiving CSL525 showed a 57% inhibition of muzzle calcification compared to vehicle-treated *abcc6^-/-^* mice. CSL525 inhibited skin calcification development by 60% in the calcinosis cutis rat model. **Conclusions:** CSL525 may prove beneficial not only in preventing the progression of cardiovascular calcification but also in treating other ectopic calcification conditions, including skin calcification associated with genetic disorders such as PXE.

## 1. Introduction

Ectopic calcification is a pathological condition characterized by the mineralization of soft tissues due to the abnormal deposition of calcium phosphate crystals in the extracellular matrix and on dead or degenerating cells [1]. This multifactorial and complex process [2] involves disturbances in calcium and phosphorus homeostasis and metabolism [3,4,5], the depletion of local or circulating crystallization inhibitors [6], extracellular matrix degradation and remodeling by metalloproteinases and proteases [7], as well as the phenotypic trans-differentiation of resident vascular smooth muscle cells into an osteoblast-like phenotype and apoptosis [8,9]. Regardless of the triggers of calcification, the final common pathway involves the deposition of solid calcium phosphate within the extracellular matrix, primarily as hydroxyapatite crystals [10,11], but also in other crystalline forms, such as whitlockite or as amorphous salts [12]. Vascular calcification is the most prominent manifestation of ectopic calcification and serves as a predictor of increased risk of cardiovascular events and mortality [12,13]. It progresses more aggressively in patients with end-stage kidney disease on dialysis compared to those not on dialysis [14] and is a major contributor to morbidity and mortality [15,16].

Ectopic calcification can also result from genetic disorders, such as generalized arterial calcification of infancy (GACI) or pseudoxanthoma elasticum (PXE). PXE is an autosomal recessive disorder that leads to hydroxyapatite deposition in the extracellular matrix of elastin-rich connective tissues. Patients with PXE develop progressive calcification in the skin, eyes, and vascular system [17,18], and no approved treatments exist for its systematic manifestations. Classic PXE forms are caused by loss-of-function pathogenic variants in the *abcc6* gene [19,20,21]. While the substrate of ABCC6 remains unknown, recent studies have demonstrated that ABCC6-dependent low plasma levels of inorganic pyrophosphate (PPi) play a key role in the pathophysiology of PXE [22]. Experimental studies have shown that targeted deletion of the mouse *abcc6* gene leads to progressive connective tissue mineralization, a hallmark feature of PXE. In addition, studies have demonstrated that mineral deposits in *abcc6^-/-^* mice consist of calcium and phosphate, which combine to form hydroxyapatite crystals similar to those found in affected human tissues [23]. For these reasons, the *abcc6^-/-^* mouse model is commonly used to study PXE.

At present, no approved therapies exist to prevent or treat vascular calcification, although various therapeutic strategies have been explored to halt or reverse its progression. Some of these treatments are repurposed from existing therapies for related conditions, such as chronic kidney disease and its associated bone mineral disorders [24]. Calcimimetics, including cinacalcet and etelcalcetide, enhance the sensitivity of calcium-sensing receptors to extracellular calcium, thereby reducing parathyroid hormone levels, which in turn can help to prevent the progression of vascular calcification [25]. Phosphate binders are used to lower serum phosphate levels and mitigate hyperphosphatemia, a key contributor to vascular calcification. Non-calcium-based phosphate binders have been associated with a reduced risk of all-cause mortality compared to calcium-based binders [26], although their effects on cardiovascular calcification compared to placebo or usual care remain uncertain [27].

Additionally, other experimental approaches that specifically target the calcification process are under investigation at different stages of research. In this context, sodium thiosulfate may prevent vascular calcification by chelating calcium and forming soluble complexes. It is used off-label in patients with calciphylaxis [28] and has also shown positive effects in inhibiting calcification in animal models [29] and non-randomized clinical studies [30], although strong supportive evidence is still lacking. Furthermore, pyrophosphate, an endogenous inhibitor of calcification, and bisphosphonates, its non-hydrolyzable analogs, are therapeutic strategies that bind calcium phosphate to inhibit hydroxyapatite formation and growth [31]. Both compounds have been shown to prevent vascular calcification in animal models [32,33,34], but pyrophosphate has a limited plasma half-life [32], and bisphosphonate may cause skeletal toxicity at doses required to inhibit vascular calcification [34,35]. Some of these small-molecule therapeutic approaches have also been explored for treating ectopic soft tissue calcification in PXE [36,37,38]. The PROPHECI trial is currently evaluating the safety and efficacy of daily oral pyrophosphate in reducing or stabilizing ectopic calcification in PXE [39].

Hexasodium fytate (CSL525, previously known as SNF472) is a small-molecule crystallization inhibitor being developed for the treatment of cardiovascular calcification-related disorders, such as calciphylaxis in dialysis patients [40]. The mechanism of action of CSL525 involves the physicochemical inhibition of the formation and growth of hydroxyapatite crystals at sites of ectopic calcification, the final common pathway in calcification-related disorders [41]. Its efficacy in inhibiting vascular calcification in vivo has been demonstrated in rat models of both non-uremic and uremic cardiovascular calcification [42]. This experimental treatment has also shown efficacy in reducing cardiovascular calcification progression in a phase II clinical trial involving patients with end-stage kidney disease undergoing dialysis [43]. The mechanism of action and therapeutical potential of CSL525 have been recently reviewed [44,45].

Although promising results in inhibiting ectopic calcification have been observed in animal models and a few placebo-controlled randomized clinical trials, a recent meta-analysis concluded that data on interventions to mitigate vascular calcification in clinical trials remain insufficient and, in some cases, conflicting [46]. These limitations underscore the need for further studies to advance the development of new therapies for conditions related to ectopic calcification.

Our hypotheses were that CSL525 could represent a therapeutic option for soft tissue calcification disorders not solely related to vascular calcification and that the efficacy of the crystallization inhibitor might depend on the nature (spontaneous or induced) and severity of the calcification. Thus, our aim was to investigate the potential of CSL525 for treating ectopic calcification disorders using in vitro and in vivo models with varying degrees of calcification. To this end, we first assessed the efficacy of CSL525 in inhibiting human vascular smooth muscle cell (hVSMC) calcification in two in vitro models, each with different calcification inducers. We then assessed its in vivo efficacy in three ectopic calcification models: zebrafish and mouse models of PXE and a rat model of calcinosis cutis. The latter resembles calciphylaxis, also known as calcific uremic arteriolopathy, which is the most severe manifestation of ectopic calcification in chronic kidney disease patients [47].

## 2. Results

### 2.1. Human Vascular Smooth Muscle Cells Culture

**Figure 1:**
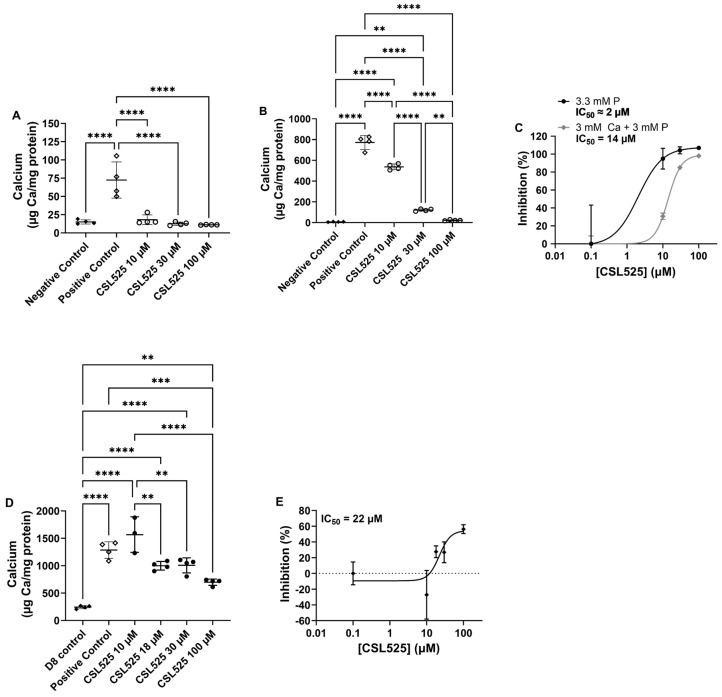
Effects of CSL525 on in vitro calcification of human vascular smooth muscle cells (hVSMCs). (**A**) Calcium content in cultures following calcification induction with 3.3 mM phosphate (P) and increasing concentrations of CSL525 for 21 days. (**B**) Calcium content in cultures following calcification induction with 3 mM P and 3 mM calcium (Ca) in the presence of increasing concentrations of CSL525 for 14 days. (**C**) Inhibition of calcification by CSL525 when added at the start of calcification induction. (**D**) Calcium content in cultures after 7 days of calcification induction with 3 mM P and 3 mM Ca, followed by CSL525 treatment initiated on day 7. (**E**) Inhibition of calcification by CSL525 when added after 7 days of calcification induction. Results are presented as mean ± SD. Statistical analysis: one-way ANOVA with Tukey’s post hoc test. (**) *p* < 0.01, (***) *p* < 0.001, (****) *p* < 0.0001.

Both calcifying media (3.3 mM phosphate and 3.0 mM phosphate + 3.0 mM calcium) induced calcification in cultures (Figure 1A,B). Calcification levels were approximately 10-fold higher in cultures treated with the combination of calcium and phosphate compared to those treated with phosphate alone. The inhibitory potency of CSL525 was influenced by the extent of calcification. In the phosphate alone model, all tested concentrations resulted in over 90% inhibition, allowing only an approximate determination of an IC_50_ of 2 µM (Figure 1C). In the combination model, a clear concentration–response curve was obtained. The lowest tested concentration of CSL525 significantly inhibited calcification by approximately 30%, with a calculated IC_50_ of 14 µM (Figure 1C).

Based on these results, we designed a third in vitro experiment using hVSMCs in which CSL525 treatment was initiated after 7 days of calcification induction. At this time point, calcification had already begun (mean of 244 µg Ca/mg protein, Figure 1D). A CSL525 concentration of 100 µM was needed to significantly inhibit calcification progression between days 8 and 15, resulting in a 56% inhibition (Figure 1E). The calculated IC_50_ for CSL525 was 22 µM, which was higher than the IC_50_ obtained when CSL525 was used as a preventive agent.

### 2.2. PXE Zebrafish Model

**Figure 2:**
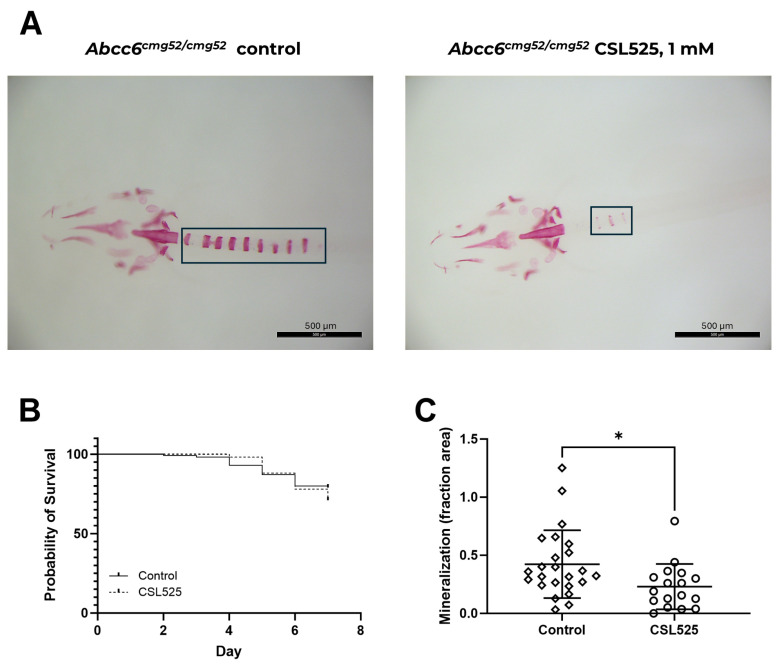
Effect of 1 mM CSL525 in an *abcc6a^cmg52/cmg52^* zebrafish model of pseudoxanthoma elasticum. A total of 100 a*bcc6a^cmg52/cmg52^* zebrafish larvae were exposed to 1 mM CSL525 at 3 days post-fertilization (dpf) for 7 days, while another group of 100 larvae was kept under the same conditions without CSL525. After 7 days of treatment (10 dpf), the fish were euthanized, fixed in 4% paraformaldehyde and stained with Alizarin Red S. (**A**) Representative images of the Alizarin Red S staining in abcc6^cmg52/cmg52^ zebrafish untreated or treated with CSL525. (**B**) Survival curves during the 7 days of study. (**C**) Mineralized fraction area of spinal mineralization. Results are presented as mean ± SD. Statistical analysis: (**A**) Kaplan–Meier survival analysis. (**B**,**C**) Mann-Whitney test. (*) *p* < 0.05.

Zebrafish embryos were divided into two groups at 3 dpf: an untreated control group and a group treated with 1 mM CSL525. By 10 dpf, overall survival in both groups was similar (69–79%, *p* = 0.2713) after seven days of treatment (Figure 2B). Genotyping confirmed that only *abcc6a^cmg52/cmg52^* larvae developed the spinal mineralization phenotype. Notably, CSL525 treatment significantly reduced mineralization by 46%, as shown in Figure 2C.

### 2.3. PXE Mouse Model

**Figure 3:**
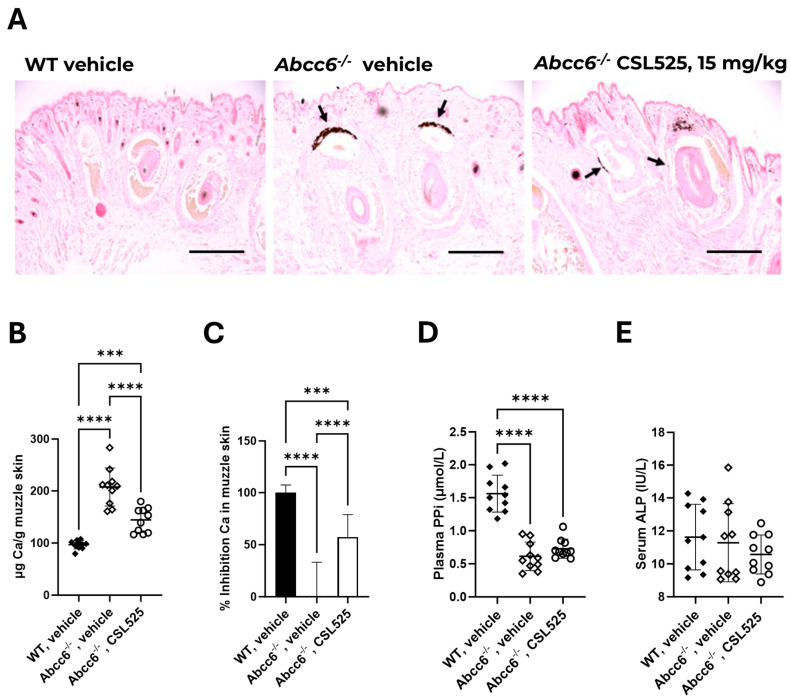
Effect of subcutaneous CSL525 at 15 mg/kg three times per week in a pseudoxanthoma elasticum a*bcc6^-/-^* mouse model. The *abcc6^-/-^* mice were subcutaneously administered CSL525 at 15 mg/kg or 0.9% NaCl thrice weekly for eight weeks. A group of C57BL/6J wild-type mice, also receiving 0.9% NaCl thrice weekly, was included in the study. Each group consisted of five males and five females. All mice were maintained on a standard diet and sacrificed at 13 weeks of age, eight weeks after treatment initiation. (**A**) Histopathology of vibrissae calcification in the mice muzzle skin (von Kossa staining). Arrows indicate ectopic calcification. Scale bar: 0.4 mm. (**B**,**C**) Calcium (Ca) content and percentage inhibition of calcification in the muzzle skin. (**D**) Plasma pyrophosphate (PPi) concentration. (**E**) Alkaline phosphatase (ALP) activity in serum. WT, wild-type C57BL/6J mice. Results are presented as mean ± SD. Statistical analysis: one-way ANOVA with Tukey’s post hoc test. (***) *p* < 0.001, (****) *p* < 0.0001.

The extent of ectopic calcification in the muzzle skin was measured using two independent assays. The left side muzzle skin was processed for histological evaluation with von Kossa staining (representative images shown in Figure 3A), while the right side was used for quantitative calcium analysis. Both the calcium-specific stain and quantitative analysis revealed robust calcification in the dermal sheath of vibrissae in vehicle-treated *abcc6^-/-^* mice (positive control) at 13 weeks of age, with 208 ± 37 µg calcium/g muzzle (Figure 3B). Ectopic calcification in positive control mice was 2.1-fold higher than in vehicle-treated WT mice (negative control, 97 ± 8 µg calcium/g muzzle), confirming the progression of ectopic calcification in *abcc6^-/-^* mice in this study.

The *abcc6^-/-^* mice receiving CSL525 at 15 mg/kg thrice weekly showed a significant reduction in muzzle skin calcification (144 ± 24 µg calcium/g muzzle) compared to vehicle-treated *abcc6^-/-^* mice. Thus, CSL525 treatment at 15 mg/kg resulted in a 57% inhibition of ectopic mineralization in the muzzle (Figure 3C).

*Abcc6^-/-^* mice exhibited reduced plasma PPi levels compared to WT mice. Plasma PPi concentrations in WT, *abcc6^-/-^*, and CSL525-treated *abcc6^-/-^* mice were determined, showing that CSL525-treated *abcc6^-/-^* mice had plasma PPi concentrations similar to those of vehicle-treated *abcc6^-/-^* mice, both approximately 41% of the levels observed in WT mice (Figure 3D). Serum alkaline phosphatase activity, a key driver of ectopic calcification, did not differ between WT, a*bcc6^-/-^*, and CSL525-treated *abcc6^-/-^* mice (Figure 3E).

Circulating levels of CSL525 10 min post dose (expected C_max_) were 5 ± 3 µM (mean ± SD).

### 2.4. Calcinosis Cutis Rat Model

**Figure 4:**
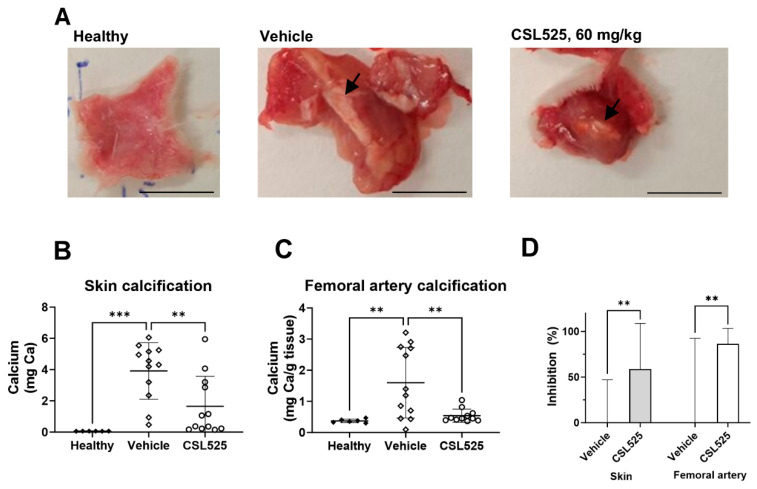
Effect of subcutaneous administration of CSL525 at 60 mg/kg once daily in a calcinosis cutis rat model. Calcinosis cutis was induced in 24 rats by sensitization with subcutaneous vitamin D (50,000 IU/kg on days 1, 2 and 3), followed by subcutaneous injections of FeCl_3_ (0.15 mg) at each of the two ventral sites in the thorax on day 4. CSL525 at 60 mg/kg (N = 12) or 0.9% NaCl (N = 12) was administered subcutaneously on a daily basis. A group of 6 healthy rats received subcutaneous 0.9% NaCl on days 1, 2, and 3, and 0.5 mL of 0.9% NaCl at each of the two ventral thoracic sites on day 4. (**A**) Representative images of the skin tissue surrounding the FeCl_3_ injection site. Arrows indicate ectopic calcification. Scale bar: 1 cm. (**B**) Calcium (Ca) content in the skin tissue surrounding the FeCl_3_ injection site. (**C**) Calcium content in femoral arteries. (**D**) Inhibition of calcification in the skin and femoral arteries by CSL525. Results are presented as mean ± SD. Statistical analysis: one-way ANOVA with Tukey’s post hoc test. (**) *p* < 0.01, (***) *p* < 0.001.

Representative images of the calcified plaque developed after FeCl_3_ injection are shown in Figure 4A. Skin calcification, expressed as mg of total calcium from the two ventral sites of the rat’s thorax, is presented in Figure 4B. Calcification values in the skin of healthy rats were low, with a mean calcium content of 0.0617 ± 0.0015 mg. In the vehicle group, after three subcutaneous administrations of vitamin D followed by a single FeCl_3_ injection at each of the two ventral thoracic sites, the mean calcium content in the collected skin at day 8 was 3.9 ± 0.5 mg. CSL525, administered subcutaneously at 60 mg/kg to rats with calcinosis cutis, inhibited skin calcification by 59% compared to the corresponding vehicle-treated group (Figure 4D).

Vitamin D induced a low degree of calcification in the femoral arteries (1.6 mg/g dry tissue in vehicle-treated rats vs. 0.4 mg/g dry tissue in healthy animals, Figure 4C), which was inhibited by 80% after treating the rats with CSL525 (Figure 4D).

Circulating CSL525 levels 20 min post dose (expected C_max_) following 60 mg/kg subcutaneous administration were 60 ± 37 µM.

## 3. Discussion

In the present article, we demonstrate that CSL525, a small molecule with previously proven efficacy in inhibiting cardiovascular calcification in human clinical trials, also prevents other forms of ectopic calcification, such as skin calcification, in animal models of PXE and calcinosis cutis.

To assess the impact of calcification severity and progression rate on the efficacy of CSL525, we tested different concentrations of the compound in vitro using two different calcifying media in hVSMCs. VSMCs are the standard cell model for studying vascular calcification in vitro. However, other cell types, such as valve interstitial cells, can be used to investigate the specific mechanisms of aortic valve calcification [48]. In VSMCs, one of the most commonly used protocols to induce calcification involves exposure to a high phosphate concentration (3.3 mM), which enters the cells through the sodium-dependent phosphate transporter 1 (Pit-1), triggering the phenotypic transformation of muscular cells into an osteoblast-like phenotype [49,50,51]. This transformation results in the deposition of calcium phosphate crystals in the extracellular matrix [52]. Other protocols induce calcification in VSMCs by combining high concentrations of both calcium and phosphate [41], which accelerates and enhances calcium phosphate crystal deposition compared to phosphate alone, likely due to the additive effects of both passive and active crystal deposition processes [53].

The differences in calcification magnitude and progression rate between both experimental models allowed us to assess calcification inhibitors in various pathological contexts. The slower progression rate in the phosphate alone protocol mimics the gradual calcification observed in spontaneous models, chronic kidney disease, and PXE. By contrast, the calcium and phosphate model is more representative of induced calcification models and pathologies with faster calcification development, such as calciphylaxis or calcinosis cutis. Our results showed that CSL525 effectively and completely inhibited the development of calcification in hVSMC cultures, regardless of severity. However, its potency was directly related to the severity of the calcification, requiring higher concentrations to achieve inhibition when calcification is more pronounced. This conclusion was further supported by the observation that both the minimum effective concentration and the IC_50_ of CSL525 were higher in the in vitro model of calcification progression, where treatment began after calcification has already initiated. Although cell viability was not assessed in this study, a previous study with a similar design (evaluating the effects of CSL525 in primary rat VSMC cultures) demonstrated that CSL525 doses up to 100 µM, which effectively inhibited calcification, did not induce apoptosis or compromise cell viability [41].

The in vivo efficacy of CSL525 was tested in three animal models of ectopic calcification. First, we tested the effects of CSL525 in a zebrafish model of PXE. This model expresses a vertebral phenotype with increased mineralization of the vertebrae [54], and it has been demonstrated to be useful for studying the efficacy of several compounds in inhibiting abnormal mineralization. These compounds include bisphosphonates (35% reduction with 100 µM etidronate and 39% with 100 µM alendronate), sodium thiosulfate (55% reduction at 20 µM), magnesium citrate (45% reduction at 10 mM), and vitamin K (42% reduction at 80 µM) [55,56]. Another therapeutic option investigated as an anti-calcification agent in PXE models is minocycline, an inhibitor of poly-ADP ribose polymerase 1 (PARP1), a pivotal member of the DNA damage response (DDR). Activation of the DDR and PARP1 signaling pathways is a hallmark of PXE pathophysiology, and it was recently demonstrated that 10 µM minocycline treatment for 7 days significantly reduced PXE zebrafish hypermineralization, with an average 60% reduction, by attenuating excessive DDR/PARP1 signaling [57]. The observed effects of these drugs are similar to those seen in PXE mouse models and patients, supporting the zebrafish model as a reliable preclinical platform for anti-mineralizing drug screening before transitioning to rodent models [58]. In the present study, CSL525 inhibited abnormal mineralization of the vertebrae by at least 46% in *abcc6a^-/-^* zebrafish larvae. However, the concentration required (1 mM) was higher than the typical effective concentrations of CSL525 reported for in vitro, ex vivo, and in vivo studies, which are typically in the low micromolar range [41,48,59,60]. This discrepancy could be attributed to the low permeability of the compound through fish skin. Phytate, the active principle of CSL525, is a polar compound with six phosphate groups attached to the inositol ring, making it hydrophilic and negatively charged. This structure limits its permeability and bioavailability through skin permeation. The subcutaneous bioavailability of CSL525 in rats has been reported to be below 10% [42], which might explain its low permeability through fish skin.

After confirming that CSL525 could inhibit the calcification associated with *abcc6* deficiency in the screening model of zebrafish, we assessed its efficacy in *abcc6* knockout mice. Ectopic calcification in *abcc6^-/-^* mice is progressive, with calcification occurring as early as 5–6 weeks postnatally [61]. By 13 weeks of age, untreated *abcc6^-/-^* control mice develop robust and quantifiable calcification in the connective tissue dermal sheath of vibrissae in the muzzle skin, which serves as an early and reliable biomarker in this PXE mouse model [61].

We conducted an 8-week efficacy study, initiating treatment in *abcc6^-/-^* mice at five weeks of age, when ectopic calcification first begins. Mice were treated with vehicle or CSL525 at 15 mg/kg via subcutaneous injections thrice weekly for eight weeks. Our results demonstrated that thrice-weekly administration of CSL525 at 15 mg/kg (total weekly dose of 45 mg/kg) effectively prevented muzzle skin calcification in *abcc6^-/-^* mice, achieving a 57% reduction in ectopic calcification. These findings suggest that CSL525 could represent a potential pharmacological approach worth further investigation for spontaneous calcification in PXE and GACI type 2, diseases for which there are currently no preventive or therapeutic options. Maximum circulating levels of CSL525 (approximately 10 min post dose) were around 5 µM, which is within the therapeutic concentration range (5–10 μM) required for in vivo efficacy in inhibiting ectopic calcification [41].

The anti-calcification effect of CSL525 was not associated with changes in plasma PPi concentrations compared to vehicle-treated knockout mice. CSL525 is a novel calcification inhibitor, and its mechanism of action involves binding to the growth sites of hydroxyapatite crystals, the primary component of calcification deposits. This binding prevents the formation and growth of these crystals in soft connective tissues prone to calcification. CSL525 operates via a different mechanism of action than other experimental therapies currently being investigated for PXE or GACI. For instance, INZ-701 is a recombinant, soluble form of the ENPP1 enzyme under development for the treatment of PXE and GACI. Subcutaneous administration of INZ-701 at 2 mg/kg every other day normalized plasma PPi levels in *abcc6^-/-^* mice and reduced calcification in the muzzle skin [62]. Jacobs et al. recently tested a more potent ENPP1 isoform, BL-1118, in the *abcc6^-/-^* mouse model. When administered once weekly, BL-1118 dose-dependently increased steady-state plasma ENPP1 activity, subsequently raising PPi levels. Subcutaneous weekly dosing of BL-1118 for ten weeks significantly reduced ectopic calcification in the muzzle skin and kidneys of *abcc6^-/-^* mice [63]. ENPP1 catalyzes the dephosphorylation of ATP to AMP and PPi, functioning downstream of ABCC6, which is responsible for transporting ATP to the extracellular space. However, increased ENPP1 activity appears to be sufficient to compensate for the ATP transport deficit, thereby restoring physiological PPi concentrations.

Inhibition of alkaline phosphatase, which hydrolyzes PPi to inorganic phosphate, has been proposed as another alternative to treat ABCC6 or ENPP1 deficiency. One study in *abcc6^-/-^* mice showed that the alkaline phosphatase inhibitor SBI-425, administered at 75 mg/kg/day, increased circulating PPi levels and inhibited muzzle skin calcification [64]. However, the same compound failed to show efficacy in an *enpp1* knockout mouse model of GACI [64]. A recent publication showed that high-dose oral minocycline supplementation (40 mg/kg/day) for 12 months in *abcc6*^−/−^ mice inhibited the development of ectopic calcification in the muzzle skin [65].

CSL525 is not the first calcification inhibitor tested in PXE mouse models. INS-3001, a phytic acid analog with the same mechanism of action as CSL525, inhibited muzzle skin calcification in *abcc6^-/-^* mice when administered subcutaneously three times per week at approximately 9 mg/kg [66]. These results support the idea that inhibiting hydroxyapatite formation and growth is a promising therapeutic strategy for PXE. Compounds that target hydroxyapatite formation may have off-target effects, potentially impacting physiological hydroxyapatite in bones. In a study where INS-3001 prevented calcification in an adenine diet-induced rat model of uremic calcification, an increase in the osteoid area was also observed in the treatment groups. Given the preserved osteoblast number and activity, this suggested an imbalance between osteoid deposition and subsequent mineralization [67]. CSL525 did not affect rat primary osteoblast differentiation in vitro, nor did it have deleterious effects on bone mineralization after 9 months of repeated dosing in Beagle dogs [41]. However, bone mineral density decreased modestly following CSL525 administration for 1 year in hemodialysis patients [68].

The PXE mouse model is characterized by low-degree calcification with slow progression. This can be observed in the results obtained in this study, where calcification in the muzzle skin of *abcc6^-/-^* mice progressed approximately 2-fold over the 8 weeks of study, which aligns with previous studies using the same model [62,63,64,66]. A similar rate of progression was observed when muzzle skin calcification was monitored until 24 weeks of age [66]. These slow-progression models represent certain genetic calcification disorders (such as PXE or GACI) and offer an advantage when evaluating less frequent dosing regimens, such as weekly or three-times-weekly administrations. However, other disorders related to skin calcification, such as calciphylaxis, exhibit faster rates of calcification progression. Therefore, to assess the efficacy of CSL525 in a high-degree and fast-progressing calcification model, we employed a calcinosis cutis model, in which the calcium content in the skin increased 100-fold in just four days. Skin calcification development in this model relies on lesions induced by oxidized iron after animals have been sensitized to calcification by vitamin D [69]. There are currently few animal models of calcinosis cutis, most of which involve high doses of vitamin D_3_ combined with a local calcification inducer. Price et al. demonstrated the efficacy of ibandronate as a calcification inhibitor in a rat model of vascular and cutaneous calcification induced by a high dose of vitamin D_3_ and local administration of FeCl_3_ (300 µg), respectively [69]. Later, Grases et al. developed a rat model of calcinosis cutis using two subcutaneous injections of 0.1% KMnO_4_ in the interscapular area. Both KMnO_4_ and FeCl_3_ act as oxidants, triggering local calcification and plaque formation. It that study, topical application of myo-inositol hexaphosphate reduced plaque formation by up to 90% [70]. In our lab, we established a rat model of calcinosis cutis based on the model by Price et al., refining the conditions to induce cutaneous calcification within 8 days using reduced doses of vitamin D_3_ and FeCl_3_ to evaluate potential inhibitors.

Despite rapid calcification progression in skin, daily CSL525 treatment achieved 60% inhibition. The efficacy of CSL525 in inhibiting severe calcification in vivo has been previously reported. Ferrer et al. demonstrated that CSL525 inhibited up to 85% of aortic calcification in a uremic rat model, in which the calcium content in the aorta increased more than 100-fold over 19 days [42]. The higher efficacy observed in that study may be attributed to the high dose administered and the use of a 100% bioavailable route of administration (50 mg/kg by intravenous infusion). In the present study, sensitization with a low dose of vitamin D (50,000 IU/kg for three days) was required to induce calcinosis cutis, as evidenced by increased calcification in the femoral arteries compared to non-sensitized rats. The calcium levels in these vessels were comparable to those observed in another low-degree calcification model induced by dietary warfarin [71]. CSL525′s inhibition of calcification in this tissue was slightly higher than in the skin, likely due to the lower degree of calcification in the femoral arteries. In support of this, CSL525 achieved similar inhibition (approximately 60%) in both the PXE and calcinosis cutis models, but it required a lower dose (15 mg/kg vs. 60 mg/kg) and less frequent administration (thrice weekly vs. daily) in the PXE model.

Although this study provides valuable insights into the role of CSL525 in the inhibition of ectopic calcification, some limitations should be considered when interpreting the results. In the zebrafish model, CSL525 absorption appeared lower than expected, requiring a high dose to achieve significant calcification inhibition. This, combined with the absence of a pharmacokinetic profile in this screening model, complicates direct translation to mammals. Other limitations include the use of a single dose regimen in each rodent study, with regimens differing between models, making direct comparisons challenging.

## 4. Materials and Methods

### 4.1. Human Vascular Smooth Muscle Cells Culture

Primary hVSMCs obtained commercially (Lonza, Bassel, Switzerland) were seeded in 12-well cell culture plates with standard DMEM medium supplemented with 20% fetal bovine serum, 1 mM sodium pyruvate, 2 mM glutamine, 100 UI/mL penicillin, 100 µg/mL streptomycin, and 0.25 µg/mL amphotericin. Cells were grown until reaching 90% confluence. Once confluent, the standard medium was replaced with one of two different calcifying media. One calcifying medium contained 3.3 mM phosphate in standard medium for three weeks, while the other contained 3.0 mM calcium and 3.0 mM phosphate in standard medium for two weeks. CSL525 was tested at concentrations of 10, 30, and 100 µM. Both experiments included a negative control (standard medium) and a positive control (corresponding calcifying medium without CSL525).

A third experiment evaluated the effectiveness of CSL525 in inhibiting calcification progression in hVSMC cultures that had already begun to calcify. In this experiment, the culture medium was replaced with calcifying medium (3 mM calcium and 3 mM phosphate in standard medium). After 7 days of exposure to calcifying medium, CSL525 treatment (at 10, 18, 30, and 100 µM) began on day 8. The culture was terminated on day 15, after 7 days of treatment with the inhibitor. One set of negative and positive controls was collected on day 8 to determine basal calcification at the time treatment began.

At the end of each experiment, the total calcium content deposited per well was determined by incubating the cells overnight (37 °C, 5% CO_2_) with 500 µL of 0.6 N hydrochloric acid (HCl), followed by collection of the supernatant. The total protein content per well was obtained by lysing the cells immediately after HCl sample collection using 500 µL of 0.1 N NaOH—0.1% sodium dodecyl sulfate (SDS).

The calcium content in the HCl samples was measured using inductively coupled plasma–optical emission spectrometry (ICP-OES, PerkinElmer Inc., Waltham, MA, USA) with 1% HNO_3_ as the carrier and radial plasma view at 315.887 nm.

The protein content was quantified using the Bradford method [72]. Bradford reagent was added to all samples and standards (bovine serum albumin) in duplicate, and the mixture was incubated for 10 min with constant agitation. Absorbance at 595 nm was measured using a microplate spectrophotometer (Agilent Technologies, Santa Clara, CA, USA).

The calcification level for each sample was expressed as µg Ca/mg protein. The percentage of inhibition was calculated for each sample using the positive control as a reference for maximum calcification. The results were analyzed using one-way ANOVA with Tukey’s post hoc test (GraphPad Prism 10.0, GraphPad Software, LLC, Boston, MA, USA). Statistical significance was set at *p* < 0.05.

### 4.2. PXE Zebrafish Model

All zebrafish were housed at the Zebrafish Core Facility of the Ghent Center for Medical Genetics under standard husbandry conditions, maintaining an outbred AB background. The zebrafish experiments were approved by the Animal Experimentation Committee of Ghent University (approval number: ECD 18–53). The *abcc6a^cmg52^*^/+^ zebrafish were in-crossed according to Van Gils et al., 2022 [55], and the resulting offspring were transferred into groups of 100 into petri dishes (16.2 mm height and 90 mm diameter) and maintained in an incubator at 28 °C. At 3 days post fertilization (dpf), one group of 100 larvae was exposed to 1 mM CSL525 for 7 days, while another group of 100 larvae was kept under the same conditions without CSL525. The treatment was added to the fish water (E3 medium) and refreshed daily throughout the study period.

**Figure 5:**
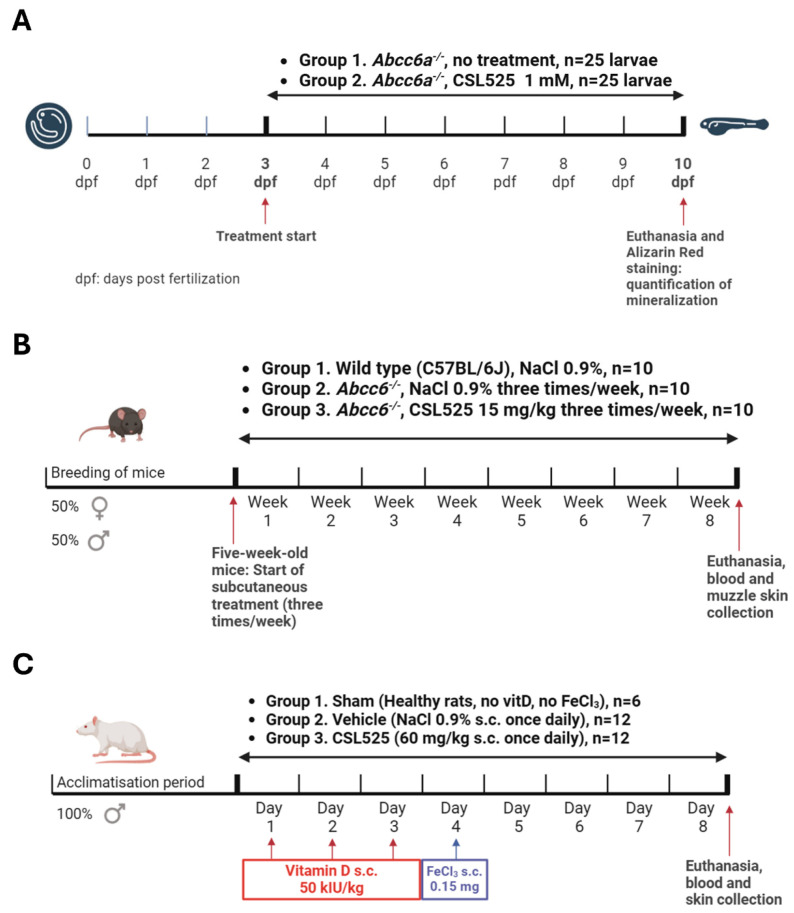
Schematic representation of the protocols used in the in vivo studies. (**A**) Zebrafish model of pseudoxanthoma elasticum (PXE). A total of 100 *abcc6a^cmg52/cmg52^* zebrafish larvae were exposed to 1 mM CSL525 at 3 days post fertilization (dpf) for 7 days, while another group of 100 larvae was kept under the same conditions without CSL525. After 7 days of treatment (10 dpf), the fish were euthanized, fixed in 4% paraformaldehyde, and stained with Alizarin Red S. (**B**) Mouse model of PXE. The *abcc6^-/-^* mice were subcutaneously administered CSL525 at 15 mg/kg or 0.9% NaCl thrice weekly for eight weeks. A group of C57BL/6J wild-type mice, also receiving 0.9% NaCl thrice weekly, was included in the study. Each group consisted of five males and five females. All mice were maintained on a standard diet and sacrificed at 13 weeks of age, eight weeks after treatment initiation. (**C**) Rat model of calcinosis cutis. Calcinosis cutis was induced in 24 rats by sensitization with subcutaneous vitamin D (50,000 IU/kg on days 1, 2 and 3), followed by subcutaneous injections of FeCl_3_ (0.15 mg) at each of the two ventral thoracic sites on day 4. CSL525 at 60 mg/kg (N = 12) or 0.9% NaCl (N = 12) was administered subcutaneously on a daily basis. A group of 6 healthy rats received subcutaneous 0.9% NaCl on days 1, 2, and 3, and 0.5 mL of 0.9% NaCl at each of the two ventral thoracic sites on day 4.

After 7 days of treatment (10 dpf), the fish were euthanized, fixed in 4% paraformaldehyde, and stained with Alizarin Red S. Digital images were captured, and the larvae were subsequently genotyped following previously established methods [73]. A schematic of the experimental protocol is shown in Figure 5A.

The results from the two groups of zebrafish receiving different treatments were analyzed using the Mann-Whitney test (GraphPad Prism 10.0, GraphPad Software, LLC, Boston, MA, USA). Statistical significance was set at *p* < 0.05.

### 4.3. PXE Mouse Model

The *abcc6* knock-out mice (*abcc6^-/-^*) on a C57BL/6J background were generated as previously described [61]. Wild-type (WT) C57BL/6J mice from The Jackson Laboratory (Bar Harbor, ME, USA) were used as controls. In this calcification prevention study, 5-week-old *abcc6^-/-^* mice were subcutaneously administered CSL525 at 15 mg/kg thrice weekly for eight weeks. Vehicle-treated WT and *abcc6^-/-^* mice served as negative and positive controls for ectopic calcification, respectively, and received subcutaneous injections of 0.9% NaCl three times per week. The injection volume in all groups was 5 mL/kg. Each group consisted of five males and five females. All mice were maintained on a standard diet and sacrificed at 13 weeks of age, eight weeks after treatment initiation. A schematic of the experimental protocol is shown in Figure 5B. All protocols were approved by the Institutional Animal Care and Use Committee of Thomas Jefferson University (protocol number 00123).

At the end of the study, animals were anesthetized by continuous inhalation of 3–4% isoflurane in an isoflurane vaporizer (Viking Medical, Artisan Technology Group, Kansas City, MO, USA) within an induction chamber at 100% oxygen and a flow rate of 1.5 L/min. Whole blood was collected via cardiac puncture 10 min after the last compound injection. Serum alkaline phosphatase activity was measured using a colorimetric kit (Abcam, Waltham, MA, USA). The PPi concentration was quantified in EDTA plasma as previously described [74,75]. Compound concentrations in EDTA plasma were analyzed by high-performance liquid chromatography with tandem mass spectrometry (HPLC-MS/MS, Waters, Milford, MA, USA) using the method described by Tur et al. for CSL525 quantification [76].

Muzzle skin biopsies (left and right sides) from euthanized mice were collected to assess the extent of ectopic calcification in the muzzle skin through two independent assays. The left side muzzle skin was processed for histological evaluation. Tissue samples were fixed in 10% phosphate-buffered formalin and embedded in paraffin. Paraffin sections (6 µm) were stained with hematoxylin and eosin or von Kossa stain using standard procedures. The right side muzzle skin biopsies were decalcified with 1.0 N HCl for 48 h at room temperature to quantify the calcium deposition. The solubilized calcium content was then determined using a colorimetric assay (Stanbio Laboratory, Boerne, TX, USA). The values of calcium content in the right muzzle skin were normalized to tissue weight (µg of calcium/g of tissue).

The results from different treatment groups of mice receiving different treatments were analyzed using one-way ANOVA with Tukey’s post hoc test (GraphPad Prism 10.0, GraphPad Software, LLC, Boston, MA, USA). Statistical significance was set at *p* < 0.05.

### 4.4. Calcinosis Cutis Rat Model

The procedures in this study were conducted in accordance with protocols approved by the Animal Experimentation Ethics Committee (CEEA) of the University of the Balearic Islands (protocol number IB 2022/05/AEXP).

A total of 30 male Sprague Dawley rats were weighed one day prior to the start of the procedure and randomized by body weight into one group of 6 animals (group 1) and two groups of 12 animals (groups 2 and 3). The thorax of the rats was shaved one day prior to the start of the procedure. Calcinosis cutis was induced in 24 rats (groups 2 and 3) by sensitization with subcutaneous vitamin D, followed by subcutaneous injections of FeCl_3_ at each of the two ventral sites in the thorax, as shown in Figure 5C. Sensitization was performed daily for three consecutive days (days 1, 2, and 3) in groups 2 and 3 by subcutaneous interscapular administration of 50,000 IU/kg vitamin D3 (cholecalciferol, Duphafral D3 1000, Zoetis, Vall de Bianya, Spain). Group 1 (healthy rats, N = 6) received subcutaneous 0.9% NaCl during these three days. The application volume in both cases was 2 mL/kg. On day 4, calcification was induced in groups 2 and 3 by two subcutaneous administration of 0.5 mL of FeCl_3_ (0.3 mg/mL) at each of the two ventral sites in the thorax (0.15 mg FeCl_3_ at each site). Group 1 received 0.5 mL of 0.9% NaCl at each of the two ventral thoracic sites. Group 2 received daily subcutaneous injections of 0.9% NaCl at an application volume of 2 mL/kg. Group 3 received daily subcutaneous administration of CSL525 at 60 mg/kg, using the same application volume.

On day 8, all animals were administered with 0.9% NaCl or CSL525 at 60 mg/kg 20–25 min before sacrifice (close to C_max_ based on rat pharmacokinetics) [42]. Blood was obtained by exsanguination of anesthetized animals (continuous inhalation of isoflurane, using an isoflurane vaporizer within an induction chamber at 3% oxygen and 1.5 L/min flow) through cardiac puncture. Plasma and serum fractions were then isolated from these blood samples.

After exsanguination, animals were euthanized by anesthetic overdose (isoflurane), and the femoral arteries, along with the calcified plaque formed at the subcutaneous FeCl_3_ injection sites (approximately 1.5 cm^2^ of skin from the injection site), were collected.

The calcium content was quantified in the skin plaques and the right and left femoral arteries. The tissues were removed at sacrifice, cleaned with 0.15 M saline, and lyophilized. The tissue dry weight was registered, and the organs were digested in a 1:1 HNO_3_:HClO_4_ mixture in a dry bath incubator for 2–4 h at 180 °C. The calcium content was quantified using ICP-OES (PerkinElmer Inc., Waltham, MA, USA).

CSL525 plasma concentrations were determined by HPLC-MS/MS (Waters, Milford, MA, USA) following the method described by Tur et al. [76].

Results from different groups were analyzed using one-way ANOVA with Tukey’s post hoc test (GraphPad Prism 10.0, GraphPad Software, LLC, Boston, MA, USA). Statistical significance was set at *p* < 0.05.

## 5. Conclusions

CSL525 can inhibit calcification in both spontaneous models, where calcification arises naturally due to genetic mutations, and induced models, where calcification is triggered by external factors. Prior evidence suggested that CSL525 can inhibit cardiovascular calcification in animal models and humans. In this article, we demonstrate that CSL525 may not only be effective in inhibiting cardiovascular calcification but also useful for treating other forms of ectopic calcification, including skin calcification in genetic disorders such as PXE. Given the variability in calcification mechanisms across different conditions, the dose and regimen of administration should be tailored to the nature (spontaneous or induced calcification), degree, and progression rate of ectopic calcification in each specific disease. Future studies, including those using escalating doses of CSL525 to characterize the dose–response curve, as well as therapeutic studies in which CSL525 is administered after calcification has already begun, may help to confirm its efficacy and potential clinical applicability in preventing or reducing pathological calcification in novel genetic models.

## Data Availability

The data presented in this study are available upon request from the corresponding author. The funding agencies had no role in the study design, data collection, analysis, or interpretation of results.
